# Functional Plasticity Coupled With Structural Predispositions in Auditory Cortex Shape Successful Music Category Learning

**DOI:** 10.3389/fnins.2022.897239

**Published:** 2022-06-28

**Authors:** Kelsey Mankel, Utsav Shrestha, Aaryani Tipirneni-Sajja, Gavin M. Bidelman

**Affiliations:** ^1^School of Communication Sciences and Disorders, University of Memphis, Memphis, TN, United States; ^2^Institute for Intelligent Systems, University of Memphis, Memphis, TN, United States; ^3^Center for Mind and Brain, University of California, Davis, Davis, CA, United States; ^4^Department of Biomedical Engineering, University of Memphis, Memphis, TN, United States; ^5^Department of Speech, Language and Hearing Sciences, Indiana University, Bloomington, IN, United States

**Keywords:** auditory learning, EEG, auditory event related potentials (ERPs), morphometry, music perception, individual differences, categorical perception (CP)

## Abstract

Categorizing sounds into meaningful groups helps listeners more efficiently process the auditory scene and is a foundational skill for speech perception and language development. Yet, how auditory categories develop in the brain through learning, particularly for non-speech sounds (e.g., music), is not well understood. Here, we asked musically naïve listeners to complete a brief (∼20 min) training session where they learned to identify sounds from a musical interval continuum (minor-major 3rds). We used multichannel EEG to track behaviorally relevant neuroplastic changes in the auditory event-related potentials (ERPs) pre- to post-training. To rule out mere exposure-induced changes, neural effects were evaluated against a control group of 14 non-musicians who did not undergo training. We also compared individual categorization performance with structural volumetrics of bilateral Heschl’s gyrus (HG) from MRI to evaluate neuroanatomical substrates of learning. Behavioral performance revealed steeper (i.e., more categorical) identification functions in the posttest that correlated with better training accuracy. At the neural level, improvement in learners’ behavioral identification was characterized by smaller P2 amplitudes at posttest, particularly over right hemisphere. Critically, learning-related changes in the ERPs were not observed in control listeners, ruling out mere exposure effects. Learners also showed smaller and thinner HG bilaterally, indicating superior categorization was associated with structural differences in primary auditory brain regions. Collectively, our data suggest successful auditory categorical learning of music sounds is characterized by short-term functional changes (i.e., greater post-training efficiency) in sensory coding processes superimposed on preexisting structural differences in bilateral auditory cortex.

## Introduction

Classifying continuously varying sounds into meaningful categories like phonemes or musical intervals enables more efficient processing of an auditory scene (Bidelman et al., 2020). Categorization of auditory stimuli is also a foundational skill for language development and is believed to arise from both learned and innate factors (Rosen and Howell, 1987; Livingston et al., 1998; Pérez-Gay Juárez et al., 2019; Mankel et al., 2020a,b). Auditory categories are further shaped by experiences such as speaking a second language (Lively et al., 1993; Escudero et al., 2011; Perrachione et al., 2011) or musical training (Bidelman et al., 2014; Wu et al., 2015; Bidelman and Walker, 2019), suggesting flexibility in categorical perception with learning. While the behavioral aspects of category acquisition are well documented, the underlying neural mechanisms and the influence of individual differences in shaping this process are poorly understood.

Characterizing the neurobiology of category acquisition is typically confounded by prior language experience and the overlearned nature of speech (Liu and Holt, 2011). For example, perceptual interference from native-language categories can impede the learning of foreign speech sounds (Guion et al., 2000; Flege and MacKay, 2004; Francis et al., 2008). Instead, non-speech stimuli (e.g., music) offer the ability to probe the neural mechanisms of nascent category learning without the potential confounds of language background or automaticity that stems from using speech materials (Guenther et al., 1999; Smits et al., 2006; Goudbeek et al., 2009; Liu and Holt, 2011; Yi and Chandrasekaran, 2016). In this regard, musical categories (i.e., intervals, chords) offer a fresh window into tabula rasa category acquisition. Indeed, non-musicians are unable to adequately categorize musical stimuli despite their exposure to music in daily life (Locke and Kellar, 1973; Siegel and Siegel, 1977; Howard et al., 1992; Klein and Zatorre, 2011; Bidelman and Walker, 2019). While several studies have assessed category learning of musical intervals, they either used highly trained listeners (Burns and Ward, 1978) or focused on different training methods that maximize learning gains (Pavlik et al., 2013; Little et al., 2019). To our knowledge, very few studies have assessed the *neural* changes associated with category learning in music.

Speech categorization is believed to emerge in the brain around N1 of the cortical event-related potentials (ERPs) and is fully manifested by P2 (i.e., ∼150–200 ms; Bidelman et al., 2013b; Ross et al., 2013; Bidelman and Lee, 2015; Alho et al., 2016; Bidelman and Walker, 2017; Mankel et al., 2020a). Fewer studies have examined the electrophysiological underpinnings of music categorization, but evidence from musicians suggests a similar neural time course (Bidelman and Walker, 2019). Functional magnetic resonance imaging (fMRI) indicates that categorization training leads to a decrease in perceptual sensitivity for within-category stimuli in auditory cortex while learning to discriminate categorical sounds shows the opposite effect—greater sensitivity to differences between stimuli (Guenther et al., 2004). Still, the majority of studies on category learning have involved speech. Speech and music categorization may invoke separate yet complementary networks in the left and right hemispheres, respectively (Desai et al., 2008; Chang et al., 2010; Liebenthal et al., 2010; Klein and Zatorre, 2011, 2015; Alho et al., 2016). Although there are likely some parallels across domains (Liu and Holt, 2011), it remains unclear whether the neuroplastic changes from rapidly learning non-speech categories such as musical intervals parallel that of speech.

More generally, auditory perceptual learning studies have reported changes in both early sensory-evoked (i.e., N1, P2) and late slow-wave ERP responses following training (Tremblay et al., 2001, 2009; Atienza et al., 2002; Tremblay and Kraus, 2002; Bosnyak et al., 2004; Alain et al., 2007, 2010; Tong et al., 2009; Ben-David et al., 2011; Carcagno and Plack, 2011; Wisniewski et al., 2020). A true biomarker of learning, however, should vary with learning performance (Tremblay et al., 2014). Because modulations in P2 amplitudes occur with mere passive stimulus exposure in the absence of training improvements, some posit P2 reflects aspects of the task acquisition process rather than training or perceptual learning, *per se* (Ross and Tremblay, 2009; Ross et al., 2013; Tremblay et al., 2014). Given the equivocal role of P2 in relation to auditory learning, we aimed to re-adjudicate whether changes in P2 scale with individual behavioral outcomes as listeners rapidly acquire novel music categories.

There is also significant variability in the acquisition of auditory categories (e.g., Howard et al., 1992; Golestani and Zatorre, 2009; Mankel et al., 2020b; Silva et al., 2020), especially for speech (Wong et al., 2007; Díaz et al., 2008; Mankel et al., 2020a; Fuhrmeister and Myers, 2021; Kajiura et al., 2021). More successful learners show greater neural activation, particularly in auditory cortex (Wong et al., 2007; Díaz et al., 2008; Kajiura et al., 2021). Such variability might be attributable to differences in the creation or retrieval of long-term memories for prototypical vs. non-prototypical sounds during learning (Golestani and Zatorre, 2009). However, we have previously shown better categorizers show efficiencies even in early sensory processing (∼150–200 ms), suggesting stimulus representations themselves are tuned at the individual level rather than later memory-related processes, *per se* (Mankel et al., 2020a).

In addition to differences in functional processing, individual categorization abilities may be partially driven by preexisting structural advantages within the brain (Ley et al., 2014; Fuhrmeister and Myers, 2021). Paralleling the left hemisphere bias for speech (Binder et al., 2004; Myers et al., 2009; Lee et al., 2012; Bouton et al., 2018), categorization of musical sounds is believed to involve a frontotemporal network in the right hemisphere, including key brain regions such as the primary auditory cortex (PAC), superior temporal gyrus (STG), and inferior frontal gyrus (IFG) (Klein and Zatorre, 2011, 2015; Bidelman and Walker, 2019; Mankel et al., 2020a; Gertsovski and Ahissar, 2022). PAC/STG size (primarily right hemisphere) has also been associated with perception of relative pitch and musical transformation judgments (Foster and Zatorre, 2010), melodic interval perception (Li et al., 2014), spectral processing (Schneider et al., 2005), and even musical aptitude (Schneider et al., 2002). To our knowledge, few studies have examined structural correlates of categorization at the individual level. In the domain of speech, faster, more successful learners of non-native phonemes exhibit larger left Heschl’s gyrus (Golestani et al., 2007; Wong et al., 2008) and parietal lobe volumes (Golestani et al., 2002). Additionally, better and more consistent speech categorizers show increased right middle frontal gyrus surface area and reduced gyrification in bilateral temporal cortex (Fuhrmeister and Myers, 2021). We thus hypothesized that successful category learning for non-speech (i.e., musical) sounds would be predicted by neuroanatomical differences (e.g., gray matter volume, cortical thickness), perhaps with a right PAC bias.

The aim of this study was to examine the functional and structural neural correlates of auditory category learning following short-term identification training of music sound categories (i.e., intervals). Musical intervals allowed us to track sound-to-label learning without the potential lexical-semantic confounds inherent to using speech materials (Liu and Holt, 2011). We measured learning-related changes in the cortical ERPs in musically naïve listeners against a no-contact control group to determine the specificity of neuroplastic effects. If rapid auditory category learning is related to enhanced sensory encoding of sound, we predicted changes in early brain activity manifesting at or before auditory object formation (i.e., prior to ∼250 ms; P2). If instead, short-term learning is associated with later cognitive processes related to decision and/or task strategy, we expected neural effects to emerge later in the ERP time course (e.g., late slow waves > 400–500 ms; Alain et al., 2007). Additionally, we anticipated successful learners would recruit neural resources in right auditory cortices, mirroring the left hemispheric specialization supporting speech categorization (Liebenthal et al., 2005; Joanisse et al., 2007; Klein and Zatorre, 2011; Bidelman and Walker, 2019). Our findings show that successful auditory category learning of musical intervals is characterized by both structural and functional differences in auditory cortex. The presence of anatomical differences along with ERP changes specific to learning suggest that the acquisition of auditory categories depend on a layering of preexisting and short-term plastic changes in the brain.

## Materials and Methods

### Participants

Our sample included *N* = 33 participants. Nineteen young adults (16 females) participated in the training task. An additional fourteen (7 females) served as a control group (data from Mankel et al., 2020a). All had normal hearing (thresholds ≤25 dB SPL, 250–8,000 Hz), were right-handed (Oldfield, 1971), and had no history of neurological disorders. Participants completed questionnaires that assessed education level, socioeconomic status (SES) (Entwislea and Astone, 1994), language history (Li et al., 2006), and music experience. Groups were comparable in age (learners: μ = 24.9 ± 4.0 years, controls: μ = 24.9 ± 1.7 years; *p* = 0.55), education (learners: μ = 18.5 ± 3.3 years, controls: μ = 17.3 ± 3.0 years; *p* = 0.32), and SES [rating scale of average parental education from 1 (some high school education) to 6 (Ph.D. or equivalent); learners: μ = 4.6 ± 1.3, controls: μ = 4.1 ± 0.6; *p* = 0.11]. All were fluent in English though six reported a native language other than English. We excluded tone language speakers as these languages improve musical pitch perception (Bidelman et al., 2013a). To ensure participants were naïve to the music-theoretic labels for pitch intervals, we required participants have no more than 3 years total of formal music training on any combination of instruments and none within the past 5 years. Critically, groups did not differ in prior music training (learners: μ = 1.1 ± 1.0 years, controls: μ = 0.6 ± 0.8 years; *p* = 0.14). All participants gave written informed consent according to protocol approved by the University of Memphis Institutional Review Board and were compensated monetarily for their time.

### Stimuli

**FIGURE 1:**
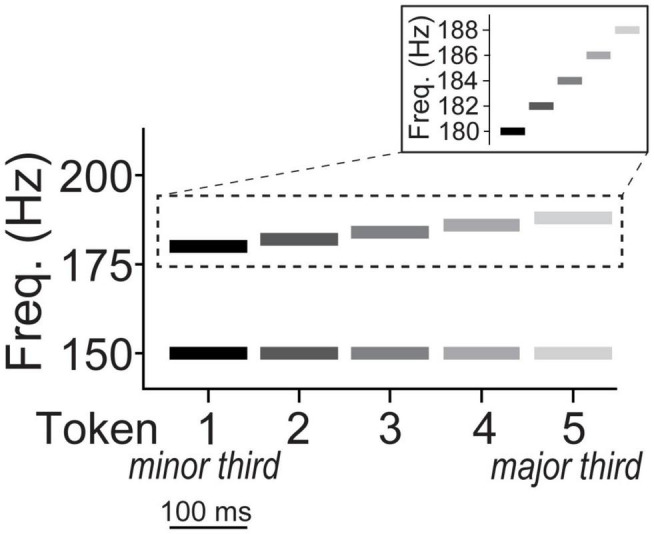
Depiction of musical interval continuum. The bass note of all five interval tokens was fixed at an F0 of 150 Hz while the upper note’s F0 ranged from 180 to 188 Hz corresponding to just intonation frequency ratios of 6:5 (minor third) and 5:4 (major third), respectively. The two notes of a given token were played simultaneously as a harmonic interval on a given trial. The individual notes of each dyad were constructed of complex tones consisting of 10 equal amplitude harmonics added in cosine phase (harmonics not shown).

We used a five-step musical interval continuum to assess category learning of non-speech sounds (Bidelman and Walker, 2017; Mankel et al., 2020b). Individual notes of each dyad were constructed of complex tones consisting of 10 equal amplitude harmonics added in cosine phase. Each token was 100 ms in duration with a 10 ms rise/fall time to reduce spectral splatter. The bass note was fixed at a fundamental frequency (F0) of 150 Hz while the upper note’s F0 ranged from 180 to 188 Hz corresponding to just intonation frequency ratios of 6:5 and 5:4, respectively (2 Hz spacing between adjacent tokens; Figure 1). The two notes of a given token were played simultaneously as a harmonic interval. Thus, the musical interval continuum spanned a minor (token 1) to major third (token 5). The minor-major third continuum was selected because these intervals occur frequently in Western tonal music and connote typical valence of “sadness” and “happiness,” respectively, and are therefore easily described to participants unfamiliar with music-theoretic labels (Bidelman and Walker, 2017). Moreover, without training, non-musicians perceive musical intervals in a continuous mode indicating they are initially heard non-categorically (Locke and Kellar, 1973; Siegel and Siegel, 1977; Burns and Ward, 1978; Zatorre and Halpern, 1979; Howard et al., 1992; Bidelman and Walker, 2017, 2019).

### Procedure

Participants were seated comfortably in an electroacoustically shielded booth. Stimuli were presented binaurally through ER-2 insert earphones (Etymotic Research) at ∼81 dB SPL. Stimulus presentation was controlled by MATLAB routed through a TDT RP2 interface (Tucker Davis Technologies). Categorization was assessed in a pre- and post-test phase. Following brief task orientation (∼2–3 exemplars), one of the five tokens was randomly presented on each trial. Participants were instructed to label the sound they heard as either “minor” or “major” *via* keyboard button press as fast and accurately as possible. The interstimulus interval was 400–600 ms (jittered in 20 ms steps) following the listener’s response to avoid anticipation of the next trial, reduce rhythmic entrainment of EEG oscillations, and to help filter out overlapping activity from the previous trial (Luck, 2014). No feedback was provided during the pre- or post-test. To reduce fatigue, participants were offered a break after each phase. Pre- and post-test procedures were similar between both the learning and control groups; the learning group received additional identification training following the pretest (see “Training Paradigm”) while the control group participants were offered a break before continuing to the posttest. Total experimental session time (including the consent process, demographics questionnaires, EEG capping, pre- & post-tests, training, etc.) was ∼2.5–3 h.

### Training Paradigm

Participants in the learning group underwent a 20-min identification training between the pre- and post-test phases. Training consisted of 500 trials, 250 presentations each of the minor and major 3rd exemplars (i.e., tokens 1 and 5), spread evenly across 10 blocks.^1^ Feedback was provided to improve accuracy and efficiency of auditory category learning (Yi and Chandrasekaran, 2016). The training procedure was conducted using E-Prime 2.0 (PST, Inc.). Listeners were successful in training if they reached ≥90% correct in at least one training block, the criterion equivalent to a trained musician’s performance on the same task (see Reetzke et al., 2018).

### EEG Acquisition and Preprocessing

EEG data were recorded using a Synamps RT amplifier (Compumedics Neuroscan) from 64 sintered Ag/AgCl electrodes at 10–10 scalp locations and referenced online to a sensor placed ∼1 cm posterior to Cz. Impedances were < 10 kΩ. Recordings were digitized at a sampling rate of 500 Hz. Preprocessing was completed in BESA Research (v7.1; BESA GmbH). Blink artifacts were individually corrected for each participant using principal components analysis (Picton et al., 2000). Bad channels were interpolated on an individual basis according to the other electrodes using spherical spline interpolation (≤ 2 channels per participant). The data were sufficiently clean following these procedures and no further trial-wise artifact rejection was necessary. Continuous data were re-referenced offline to the common average reference, filtered from 1 to 30 Hz (4th-order Butterworth filter), baselined to the prestimulus interval, epoched from −200 to 800 ms, and averaged across trials to compute ERPs for each token per electrode.

### MRI Segmentation and Volumetrics

A total of 12 out of 19 learning group participants returned on a separate day for structural MRI scanning. 3D T1-weighted anatomical volumes were acquired on a Siemens 1.5T Symphony TIM scanner (tfl3d1 GR/IR sequence; TR = 2,000 ms, TE = 3.26 ms, inversion time = 900 ms, phase encoding steps = 341, flip angle = 8°, FOV = 256 × 256 acquisition matrix, 1.0 mm axial slices). Scanning was conducted at the Semmes Murphey Neurology Clinic (Memphis, TN). All MRI T1-weighted images were corrected for inhomogeneities using an N4 bias field correction algorithm and registered to MNI ICBM 152 T1 weighted atlas with 1 × 1 × 1 mm^3^ isometric voxel size using affine transformation with 12 degrees of freedom (Dierks et al., 1999; Scott et al., 2014). The inverse transformation matrix was computed and applied to the brain mask in atlas space to create a mask in subject space (i.e., each subject’s original image space) for skull removal (Evans et al., 1993). An LPBA40 T1 weighted atlas with 2 × 2 × 2 mm^3^ voxel size was then used to register the images and remove the cerebellum using the atlas cerebrum mask and following the same process performed in subject space as explained above (Shattuck et al., 2008). After skull removal and cerebrum extraction, an AAL3 T1 weighted atlas with 1 × 1 × 1 mm^3^ voxel size that provides parcellation of a large number of brain regions was used for extracting gray matter volume in certain regions of interest (ROIs) for each participant (Rolls et al., 2020). All of the MRI pre-processing analyses were performed using in-house script written in Python^2^ using the ANTs library (Avants et al., 2009).

### Data Analysis

#### Behavioral Data

Identification curves were fit with a two-parameter sigmoid function *P* = 1/[1 + *e*^–β1(^*^x^*^–β0)^], where *P* describes the proportion of trials identified as major, *x* is the step number along the stimulus continuum, β_0_ is the locus of transition along the sigmoid (i.e., categorical boundary), and β_1_ is the slope of the logistic fit. Larger β_1_ values reflect steeper psychometric functions and therefore better musical interval categorization performance. Reaction times (RTs) were computed as the listeners’ median response latency for the ambiguous (i.e., token 3) and prototypical tokens (i.e., mean[tokens 1 and 5]; see “ERP Data”), after excluding outliers outside 250–2,500 ms (Bidelman et al., 2013b; Bidelman and Walker, 2017; Mankel et al., 2020a). As an index of training success, accuracy was calculated in the learning group as the average percent correct identification across all training trials.

#### Event-Related Potential Data

For data reduction purposes, we analyzed a subset of electrodes from a frontocentral cluster (mean of F1, Fz, F2, FC1, FCz, FC2) where categorical effects in the auditory ERPs are most prominent at the scalp (Bidelman et al., 2013b,^2014^; Bidelman and Lee, 2015; Bidelman and Walker, 2017). Peak latencies and amplitudes were quantified for P1 (40–80 ms), N1 (70–130 ms), and P2 (140–200 ms). The mean amplitude was also measured for slow wave activity between 300 and 500 ms, given prior work suggesting rapid auditory learning effects in this later time frame (Alain et al., 2007, 2010).

We also quantified neural responses at T7 and T8 to assess hemispheric lateralization. Previous work has shown neural response differences measured from these electrodes following rapid perceptual learning of concurrent speech vowels (Alain et al., 2007). For these analyses, we computed difference waves derived between the ambiguous and prototypical tokens (ΔERP = mean[ERP_*Token*1_ & ERP_*Token*5_] − ERP_*Token*3_) for both the pre- and post-test (see Mankel et al., 2020a). Larger ΔERP values indicate stronger differentiation of category ambiguous from category prototype sounds and thus reflect the degree of “neural categorization” in each hemisphere.

#### MRI Data

Each participant’s MRI images were registered to the AAL3 atlas, ROI masks were transformed to subject space, and ROI volumes were then calculated (cm^3^) (see Supplementary Figures 3, 4 for individual subject images and ROI localization). Atlas registration was confirmed using SPM12 toolbox in MATLAB (Penny et al., 2011). Cortical thickness was examined using a diffeomorphic registration based cortical thickness (DiReCT) measure (Das et al., 2009). We used the OASIS atlas (Marcus et al., 2009) for the computation of cortical thickness because it provides four brain segmentation priors for parcellating cerebrospinal fluid (CSF), cortical gray matter, white matter, and deep gray matter. 3D cortical thickness maps for each subject were computed based on these priors. Thickness maps were then multiplied with the AAL3 atlas (converted to subject space) to compute the cortical thickness of each brain region mapped to their corresponding labels. Finally, the mean, standard deviation, and range of the cortical thickness measurements along with the surface area and volume of the cortical regions were computed for each ROI. Volumetrics were normalized to each participant’s total intracranial brain volume to control for artificial differences across individuals (e.g., head size; Whitwell et al., 2001). To test for hemispheric differences specific to auditory neuroanatomic measures, we restricted ROI analysis to bilateral Heschl’s gyrus (Rolls et al., 2021).

### Statistical Analysis

Unless otherwise noted, ERPs were analyzed using generalized linear mixed-effects (GLME) regression models in SAS (Proc GLIMMIX; v9.4, SAS Institute, Inc.) with subjects as a random factor and fixed effects of training phase (two levels: pretest vs. posttest), stimulus token (two levels: tokens 1 and 5 vs. 3) and behavioral performance [identification slopes or training accuracy (learning group only); continuous measures]. We also included the interaction of phase and behavioral performance to investigate whether brain-behavior correspondences change after training. The behavioral GLME models included RTs or identification slopes as dependent variables, main effects and interactions between phase and group (two levels: control vs. learning), and an additional main effect of token in the RT model (slopes are token-independent). For the MRI data, the GLME models incorporated main effects and interactions between neuroanatomical measurements (i.e., cortical thickness or normalized gray matter volume) and phase to determine whether brain structure predicts training gains in categorization performance (i.e., dependent variable: identification slopes). We used a backward selection procedure to remove non-significant variables and report final model results throughout. *Post hoc* multiple comparisons were corrected using Tukey adjustments. Identification function slopes (β_1_) were square root transformed to improve normality and homogeneity of variance. Demographic variables were analyzed using Wilcoxon-Mann-Whitney and Fischer’s exact tests due to non-normality. An *a priori* significance level was set at α = 0.05. Conditional studentized residuals (| SR| > 2), Cook’s D (> 4/*N*), and covariance ratios (< 1) were used to identify and exclude influential outliers.

## Results

### Training Results

**FIGURE 2:**
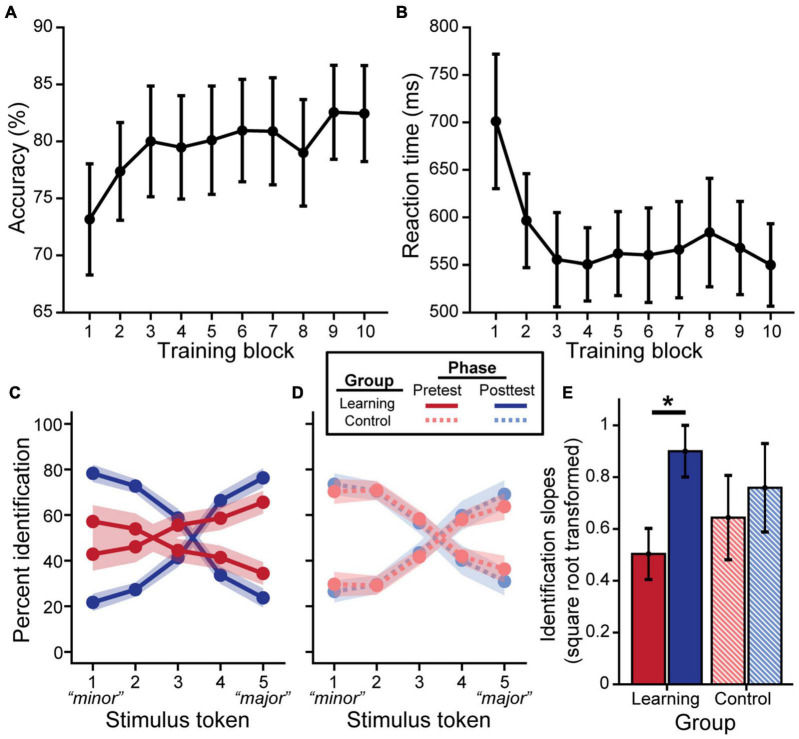
Behavioral categorization improves following rapid auditory training. Brief major/minor categorization training yields an increase in accuracy **(A)** and decrease in reaction time **(B)** across blocks. Pretest and posttest psychometric identification functions for the learning group **(C)** show stronger categorization for musical intervals after training (excluding data from *n* = 5 non-learners); performance was identical pre- to post-test for control listeners **(D)**. Slopes were square-root transformed for statistical analysis **(E)**. Error bars/shading = ± 1 SE. *p < 0.05.

Behavioral training outcomes are plotted in Figure 2. On average, participants in the learning group improved in accuracy [Figure 2A; *F*_(9, 158)_ = 2.05, *p* = 0.038] and exhibited faster RTs [Figure 2B; *F*_(9, 158)_ = 2.74, *p* = 0.005] over the course of training. Training was highly effective; most individuals averaged > 80–90% identification accuracy across the 10 blocks (i.e., the approximate performance of a musician on the same task; data not shown). *N* = 5 “non-learners” had training accuracies that did not reach the 90% criterion threshold in a block (see “Training Paradigm”) with averages remaining near chance performance (i.e., average training accuracies of 46, 46.8, 50.6, 57.8, and 68%, respectively). Consequently, these individuals were removed for all subsequent analysis. *Post hoc* analyses revealed RTs became faster following the third training block (all *p*’s < 0.05). Similarly, listeners’ identification was more accurate starting at the 9th training block compared to the first block [block 9 vs. 1: *t*_(158)_ = 3.44, *p* = 0.025; block 10 vs. 1: *t*_(158)_ = 3.40, *p* = 0.028].

### Behavioral Categorization Following Training

We then assessed training-related improvements in categorization *via* listeners’ identification of the musical interval continuum. We found a group × phase interaction for identification slopes [*F*_(1, 26)_ = 4.93, *p* = 0.035]. Importantly, control and learning groups did not differ at pretest (Figures 2C–E; *t*_26_ = −0.72, *p* = 0.48), suggesting common baseline categorization. Critically, *post hoc* analyses revealed that identification slopes were steeper at posttest for successful learners (Figure 2E; *t*_26_ = 4.42, *p* < 0.001), whereas performance remained static in the control group (*t*_26_ = 1.28, *p* = 0.21). Comparison of the probability density functions between groups of the pre- to post-test difference in slopes also suggested greater improvement in slopes for the learning group compared to the control group (see 1.1 Learning-Related Behavioral Categorization Changes in Supplementary Material and Supplementary Figure 1). For learners, in addition to training gains [main effect of phase: *F*_(1, 13)_ = 11.65, *p* = 0.005], achieving better accuracy during training was associated with steeper identification functions overall [*F*_(1, 13)_ = 8.58, *p* = 0.012]. RTs only showed an effect of phase [*F*_(1, 81)_ = 10.72, *p* = 0.002; group × phase: *F*_(1, 81)_ = 0.03, *p* = 0.856], but a trend for a group × phase interaction was also observed after removal of a single influential outlier from the learning group [i.e., in addition to the prior removal of non-learners; see “Training Results”; *F*_(1, 78)_ = 3.98, *p* = 0.050; phase: *F*_(1, 78)_ = 9.31, *p* = 0.003]. Whereas the control group achieved faster RTs at posttest [*t*_(78)_ = −3.64, *p* < 0.001], RTs remained constant in the learning group [*t*_(78)_ = −0.73, *p* = 0.466].

### Electrophysiological Results

**FIGURE 3:**
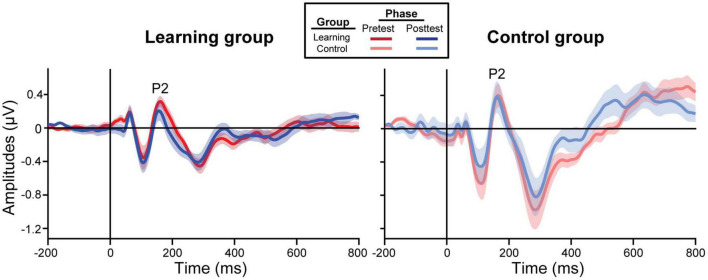
Grand average ERP waveforms collapsed across all tokens from the frontocentral electrode cluster (mean F1, Fz, F2, FC1, FCz, FC2). The learning group (left) underwent brief identification training whereas the control group (right) did not. Shading = ± 1 SE.

ERP waveforms are shown per group and experimental phase in Figure 3 (pooling all tokens). For the learning group, we found a training accuracy × phase interaction in P2 [*F*_(1, 39)_ = 5.77, *p* = 0.021] and P1 amplitudes [*F*_(1, 39)_ = 11.29, *p* = 0.002]; better performance during training was associated with decreased amplitudes in the posttest but not the pretest [P2: *t*_(39)_ = −2.71, *p* = 0.010; P1: *t*_(39)_ = −2.72, *p* = 0.010]. All other ERP comparisons with training accuracy were not significant.

**FIGURE 4:**
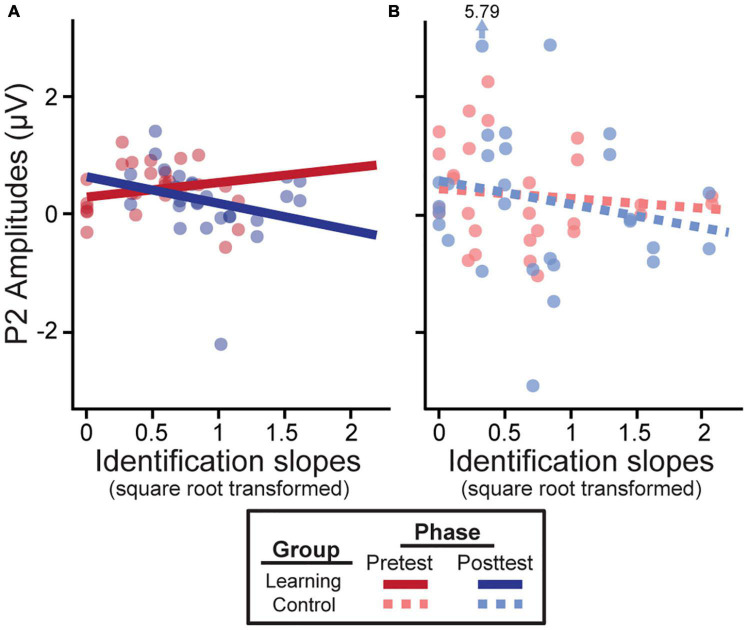
Neural amplitudes scale with behavioral outcomes in the learning group **(A)** but not the control group **(B)**. Better posttest categorization (i.e., steeper identification slopes) is associated with a decrease in P2 amplitudes. Identification slopes were square-root transformed for statistical analysis. Data points indicate individual subjects (collapsed across tokens 1 & 5 and 3). Arrow/value mark an outlier (which did not alter results).

In learners, we found an identification slopes × phase interaction for P2 amplitudes [*F*_(1, 38)_ = 4.16, *p* = 0.048]; steeper (i.e., more categorical) posttest identification slopes were associated with a decrease in neural activity after training (Figure 4A). Main effects of slope [*F*_(1, 39)_ = 8.46, *p* = 0.006] and phase [*F*_(1, 39)_ = 6.26, *p* = 0.017] were also found for the slow wave (300–500 ms). Critically, these brain-behavior relationships were specific to learners and were not observed in the control group (Figure 4B; all *p*-values > 0.05).

**FIGURE 5:**
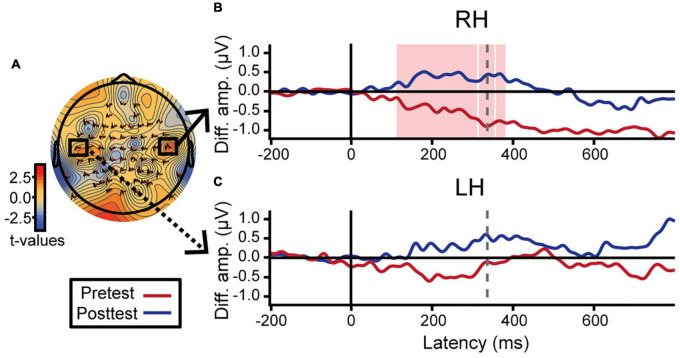
Neuroplastic changes following auditory categorical learning of music intervals are biased toward right hemisphere. Only data for the learning group is shown. **(A)** Topographic statistical map at t = 336 ms (dotted gray line in **B,C**) where pre- to post-test changes in categorical coding is maximal over the right hemisphere. Electrodes T7 and T8 are bordered by black squares. **(B,C)** Difference wave amplitudes [diff. amp.; i.e., mean(token 1 & 5) − token 3] indexing categorical neural coding (see text). An increase in neural categorization after training occurs over right (**B**; electrode T8) but not left hemisphere (**C**; electrode T7). The red shaded region in B indicates a significant effect of phase in the exploratory *t*-test (i.e., uncorrected). Average amplitudes were extracted from this time window (112–356 ms) for both hemispheres and subjected to more stringent statistical analyses (see text).

Hemispheric asymmetries were assessed *via* difference waveforms computed as the difference in voltage between brain responses to tokens 1 and 5 vs. the midpoint token 3 [i.e., mean (ERP_*Token*1_ & ERP_*Token*5_) − ERP_*Token*3_] (Bidelman and Walker, 2019). Greater difference wave values indicate stronger neural differentiation of category prototype from category ambiguous sounds, respectively, and thus index the degree of “neural categorization” in each hemisphere. This analysis focused on electrodes T7 and T8 located over the left and right temporal lobes, respectively. We used a running paired *t*-test to evaluate training effects in a point-by-point manner across the ERP time courses (BESA Statistics, v2; Figure 5). This initial, data-driven method was applied in an exploratory manner (i.e., uncorrected) to identify time windows when category encoding effects were strongest after training. In learners, category differentiation was modulated by learning 112–356 ms after stimulus onset over electrode T8 (right hemisphere; Figure 5B). Guided by these results, we then extracted average amplitudes within this time window for both the pre- and post-test and ran a more stringent (i.e., corrected for multiple comparisons) three-way mixed model ANOVA (group, identification slopes, phase). The group × slope interaction was significant for electrode T8 [*F*_(1, 23)_ = 7.86, *p* = 0.010] after removing two influential outliers (one from each group). *Post hoc* analyses revealed that for learners, steeper identification slopes predicted larger (i.e., more categorical) responses over the right hemisphere [*t*_(23)_ = 2.49, *p* = 0.021]. This brain-behavior relationship was not observed in controls nor over the left hemisphere (*p*-values > 0.05; Figure 5C). Complementary analyses of global field power similarly revealed a greater pre- to post-test change in neural activation over the right hemisphere temporal electrodes compared to the left hemisphere (Supplementary Figure 2). These data suggest a right hemisphere bias in neural mechanisms supporting category learning of musical intervals.

### Exploratory Neuroanatomical Results

**FIGURE 6:**
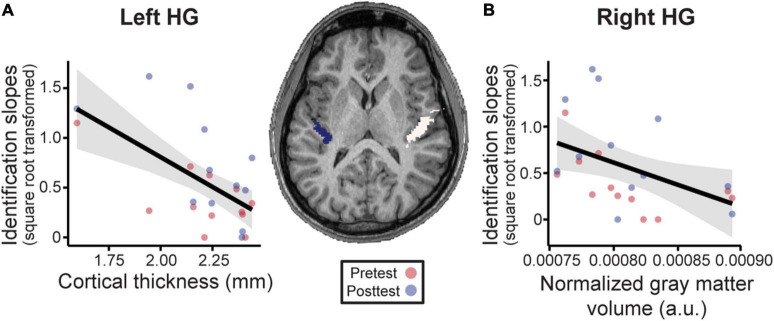
Neuroanatomical measures in Heschl’s gyrus (HG) predict behavioral categorization performance in the learning group (Center). MRI image from a representative subject with left and right HG shown in blue and white, respectively. See sSupplementary Figures 3, 4 for individual MRI images of all subjects. **(A)** In left HG, larger cortical thickness is associated with poorer categorization. **(B)** Similarly, larger normalized gray matter volumes in right HG (normalized to each individuals’ total brain volume) were associated with poorer behavioral categorization. Data points indicate individual subject identification slopes (values are square-root transformed). a.u. = arbitrary units. Shading = 95% CI.

Having established that musical interval learning leads to functional lateralization, we were interested in evaluating whether preexisting structural asymmetries (i.e., gray matter volume, cortical thickness) of Heschl’s gyrus (HG) were also associated with successful category learning. Gray matter volume was normalized according to each individual’s total brain volume for ease of inter-subject comparisons (raw data mean±SD [range] cm^3^—left: 0.80±0.05 [0.73–0.86]; right: 0.93±0.08 [0.84–1.06]; total brain volume: 1143.80±81.81 [1030.53–1302.40]). Volumetric analyses revealed that normalized gray matter volumes were larger on average in the right compared to left HG [Figure 6, center; *t*_(11)_ = 12.36, *p* < 0.001]. The interaction of phase and structural measures were not significant for identification slopes. However, phase was kept in the models to isolate the relationship between structural HG measures and behavior after factoring out training effects. Smaller normalized gray matter volumes in right HG were associated with stronger categorization overall [*F*_(1, 11)_ = 5.80, *p* = 0.035, after accounting for effects of phase; Figure 6A]. Meanwhile, thinner cortical thickness of left HG corresponded to better identification slopes [Figure 6B; *F*_(1, 11)_ = 15.07, *p* = 0.003, after accounting for effects of phase]. Cortical thicknesses and normalized gray matter volumes did not correlate with each other for either right or left HG suggesting these volumetrics provided independent measures of the anatomy (all *p*-values > 0.05). Taken together, these results indicate that preexisting differences in bilateral HG structure predict individual categorization performance.

## Discussion

By measuring multichannel EEGs and brain volumetrics during a short term auditory category learning task, our data reveal four primary findings: (i) rapid label learning of musical intervals emerges very early in the brain (∼150–200 ms, P2 wave), (ii) these ERP signatures decrease with more successful learning suggesting more efficient neural processing (i.e., reduced amplitudes) after training; (iii) neuroplastic changes in categorizing musical sounds are stronger in right hemisphere, and (iv) smaller and thinner auditory cortical regions predict better categorization performance. Successful category learning is therefore, characterized by increased functional efficiency of sensory processing, whereas better categorization performance (but not category learning) is associated with preexisting structural advantages within auditory cortex.

### Functional Correlates of Auditory Category Learning

Our data suggest musical interval category acquisition is associated with changes in ERP P2. The functional significance of P2 is still poorly understood (Crowley and Colrain, 2004). Experience-dependent neuroplasticity in P2 has been interpreted as reflecting enhanced perceptual encoding and/or auditory object representations (Garcia-Larrea et al., 1992; Shahin et al., 2003; Ross et al., 2013; Bidelman et al., 2014; Bidelman and Lee, 2015), improvements in the task acquisition process (Tremblay et al., 2014), reallocation of attentional resources (Alain et al., 2007), increased inhibition of task-irrelevant signals (Sheehan et al., 2005; Seppanen et al., 2012), or mere stimulus exposure (Sheehan et al., 2005; Ross et al., 2013). Here, we demonstrate early ERP waves including P1 (∼40–80 ms) as well as P2 (∼150–200 ms) closely scale with behavioral learning. While our experimental design does not permit a deeper probe into the listening strategies employed by the participants that resulted in improved categorization performance, our results demonstrate that the process of learning to map musical sounds (i.e., intervals) to categorical labels is associated with changes in sensory encoding responses in the brain. Moreover, these neuroplastic effects are surprisingly fast, occurring rapidly within only 20 min of training. Our findings parallel visual category learning where changes in the visual-evoked N1 and late positive component signal successful learning (Pérez-Gay Juárez et al., 2019). Our results also align with previous studies using various auditory training tasks including speech (Tremblay et al., 2001, 2009; Tremblay and Kraus, 2002; Alain et al., 2007, 2010; Ben-David et al., 2011) and non-speech sounds (Atienza et al., 2002; Bosnyak et al., 2004; Tong et al., 2009; Wisniewski et al., 2020) suggesting P2 indexes auditory experience that reflects learning success and is not simply a product of the task acquisition process (cf. Tremblay et al., 2014) or repeated stimulus exposure (Sheehan et al., 2005; Ross and Tremblay, 2009; Ross et al., 2013). The lack of clear neural effects in control listeners further rules out exposure or repetition effect accounts of our data.

In this study, successful learning (i.e., both training accuracy and identification function slopes) was characterized by a *reduction* in ERP amplitudes after training. The specific direction of P2 modulations varies across experiments with some reporting an increase in evoked responses with learning (Tremblay et al., 2001; Atienza et al., 2002; Bosnyak et al., 2004; Sheehan et al., 2005; Tong et al., 2009; Carcagno and Plack, 2011; Ross et al., 2013; Wisniewski et al., 2020) and others a decrease (Zhang et al., 2005; Alain et al., 2010; Ben-David et al., 2011). As suggested by Alain et al. (2010), such discrepancies could be related to the task (e.g., active task vs. passive recording), the stimuli (e.g., speech vs. non-speech), the rate of learning among the participants, or even the rigor of training paradigm. Studies reporting enhanced P2 often included multiple days of training or recorded ERPs during passive listening (Tremblay et al., 2001; Atienza et al., 2002; Bosnyak et al., 2004; Ross et al., 2013; Seppanen et al., 2013; Wisniewski et al., 2020). Long-term auditory experiences (e.g., music training, tone language expertise) have also been associated with enhanced P2 during active sound categorization (Bidelman et al., 2014; Bidelman and Alain, 2015; Bidelman and Lee, 2015) as well as learning (Shahin et al., 2003; Seppanen et al., 2012, 2013). The ERP decreases we find in successful learners are highly consistent with single-session, rapid learning experiments demonstrating greater efficiency of sensory-evoked neural responses during active task engagement (Guenther et al., 2004; Alain et al., 2010; Ben-David et al., 2011; Sohoglu and Davis, 2016; Pérez-Gay Juárez et al., 2019). Consequently, our results reinforce notions that the P2 is a biomarker of learning to classify auditory stimuli and map sounds to labels.

On the contrary, decreased neural activity might reflect other aspects of the task, including arousal and/or fatigue (Näätänen and Picton, 1987; Crowley and Colrain, 2004). However, decreased neural activity from these factors would have been expected in both groups due to the similar task constraints on all participants. If better learners simply sustain arousal more effectively through posttest, we would have also expected faster RTs. Rather, our data suggest decreases in activation meaningfully reflect music category learning (Gertsovski and Ahissar, 2022), paralleling findings with speech (Guenther et al., 2004). Alternatively, given modulations in both P2 and slow wave activity, a separate but overlapping processing negativity within this timeframe cannot be ruled out. Negative processing components have been associated with early auditory selection and attention (Hillyard and Kutas, 1983; Näätänen and Picton, 1987; Crowley and Colrain, 2004) and may therefore be another target for learning processes.

### Hemispheric Lateralization and Music Categorization

Our findings show that acquiring novel categories for musical intervals predominantly recruits neural resources from the right auditory cortex, complementing the left hemisphere bias reported for speech categorization (Zatorre et al., 1992; Golestani and Zatorre, 2004; Liebenthal et al., 2005, 2010, 2014; Desai et al., 2008; Myers et al., 2009; Chang et al., 2010; Alho et al., 2016). Specifically, we observed enhanced neural categorization over the right hemisphere in more successful learners. These findings support long-standing notions about lateralization for speech vs. music categorization in the brain (Zatorre et al., 1992; Desai et al., 2008; Chang et al., 2010; Liebenthal et al., 2010; Klein and Zatorre, 2011, 2015; Alho et al., 2016; Bouton et al., 2018; Bidelman and Walker, 2019; Mankel et al., 2020a). Our data parallel a study by Gertsovski and Ahissar (2022) where learning to categorize relative pitches was associated with a decrease of neural activation in right PAC as well as bilateral STG and left posterior parietal cortex. Superior music categorization in both trained musicians (Klein and Zatorre, 2011, 2015; Bidelman and Walker, 2019) as well as musically adept non-musicians (Mankel et al., 2020a) has been associated with right temporal lobe functions. We thus provide new evidence that even brief, 20-min identification training is sufficient to induce neural reorganization in the right hemisphere circuity that subserves auditory sensory coding and classification of musical stimuli.

### Neuroanatomical Correlates of Auditory Category Learning

Our MRI results indicate that individual variation in structural measures (gray matter volume, cortical thickness) within Heschl’s gyrus also predict behavioral categorization performance beyond mere training effects. Because MRIs were available for only 63% (12/19) of individuals from the learning group (and none in the control group), the findings reported here should be considered exploratory. Additional research is needed to verify the anatomical trends we see in our data. Brain structure is influenced by genetic, epigenetic, and experiential factors (Zatorre et al., 2012). Thus, it is often difficult to know whether anatomical differences are innate or experience-driven, but structural measures are presumed to be more stable and less plastic than functional responses (e.g., ERPs) (Golestani, 2012). Anatomical variability in auditory cortex has been related to learning rate and attainment for foreign speech sounds (Golestani et al., 2007), linguistic pitch patterns (Wong et al., 2008), and melody discrimination (Foster and Zatorre, 2010) as well as native speech categorization (Fuhrmeister and Myers, 2021). Consistent with this prior work on speech, our findings suggest that individual differences in music category perception and functional plasticity are influenced by anatomical predispositions within auditory cortex—that is, a layering of both nature and nurture.

It is often assumed larger morphology within a particular brain area yields better computational efficiency (i.e., “bigger is better”; Kanai and Rees, 2011). For example, faster, more successful learners of non-native speech sounds show more voluminous primary auditory cortex and adjacent white matter in left hemisphere (Golestani et al., 2002, 2007; Wong et al., 2008). Relatedly, expert listeners (i.e., musicians) show increased gray matter volumes and cortical thickness in PAC (Schneider et al., 2002; Gaser and Schlaug, 2003; Bermudez et al., 2009; Seither-Preisler et al., 2014; Wengenroth et al., 2014; de Manzano and Ullen, 2018). Instead, our data show the opposite pattern with regard to non-speech (i.e., musical interval) category learning. To our knowledge, only one study has shown correspondence between decreased gyrification in temporal regions and improved consistency in speech categorization behaviors (Fuhrmeister and Myers, 2021). Similarly, smaller gray matter volume in STG has been linked to improvements in speech and cognitive training (Takeuchi et al., 2011a,b; Maruyama et al., 2018). Additionally, the trend for reduced cortical thickness in better categorizers is consistent with previous research showing individual differences in regions of reduced cortical thickness along HG (Zoellner et al., 2019). Thus, it seems “less is more” with respect to the expanse of auditory anatomy and certain aspects of listening performance. However, future research is needed to clarify the relationships between macroscopic gray and white matter volumes measured by MRI, neuronal microstructures, and their behavioral correlates.

### Limitations

While the relationship between neural responses and individual learning performance suggests a role for P2 as an index of learning, it remains possible that some of the neural differences observed between the two groups is confounded by the experimental design. Relative to the control group, our learning group received approximately 20 additional minutes of exposure to the musical intervals during training. Increased familiarity with the musical intervals may have led to decreased ERP amplitudes in the learning group. Previous research has suggested that exposure to auditory stimuli is sufficient to induce changes in neural responses (Sheehan et al., 2005; Ross and Tremblay, 2009; Ross et al., 2013). Additionally, procedural learning is confounded with perceptual learning in this study design (Maddox and Ashby, 2004). However, we have argued above that the relationship between ERP responses and individual learning performance (i.e., accuracy and identification slopes in the learning group) suggests these neural pre- to post-test neural changes are more than simply exposure (see “Functional Correlates of Auditory Category Learning”). These effects also occur in waves that localize to auditory-perceptual areas and well before motor responses, more indicative of rapid perceptual learning due to training in our study. Future research employing an active control group, where listeners hear the same number of musical intervals but train on an unrelated task, or a passive control group with identical stimulus exposure as the learning group would be particularly useful in ruling out these potential confounds. Other modifications to the study design, such as additional training time rather than a rapid learning paradigm, might lead to more exaggerated behavioral differences between the learning and control groups (Figure 2E) and/or different brain-behavior associations altogether (e.g., enhanced neural responses; see “Functional Correlates of Auditory Category Learning”).

Although the use of musical interval categories was intentional to avoid possible confounds of language background on (novel) speech learning, it remains an open question whether our results complement category learning in other speech and non-speech domains. Our results suggest promising parallels with speech categorization and learning (Alain et al., 2010; Liebenthal et al., 2010; Bidelman et al., 2013a; Ross et al., 2013), but further research is needed to determine the domain-specificity and generality of these neural processes. Additionally, the likelihood of distributed sources outside of auditory cortex contributing to the generation of the P2 response (Crowley and Colrain, 2004; Ross and Tremblay, 2009) makes it difficult to directly relate individual differences in ERPs to our PAC neuroanatomical measures. The relationships between behavioral performance and both functional and structural measures suggest bilateral auditory cortices play a role in category learning. However, future analyses could utilize source localization techniques to more specifically determine where changes occur in the brains that predict successful category learning outcomes.

## Conclusion

We demonstrate that rapid auditory category learning of musical interval sounds is characterized by increased efficiency in sensory processing in bilateral, though predominantly right, auditory cortex. The relationship between better behavioral gains in identification performance and the ERPs corroborate P2 as an index of auditory experience and a biomarker for successful perceptual learning. The right hemisphere dominance supporting music category learning complements left hemisphere networks reported for speech categorization. These short-term functional changes can be superimposed on preexisting structural differences in bilateral auditory areas to impact individual categorization performance.

## Data Availability Statement

The raw data supporting the conclusions of this article will be made available without undue reservation. Requests for data and materials should be directed to GB, gbidel@indiana.edu.

## Ethics Statement

The studies involving human participants were reviewed and approved by University of Memphis Institutional Review Board. The patients/participants provided their written informed consent to participate in this study.

## Author Contributions

KM and GB contributed to the conception and design of the study. KM collected the data. KM and US conducted data analysis. AT-S and GB advised on the analyses. All authors contributed to the writing and revision of the manuscript.

## Conflict of Interest

The authors declare that the research was conducted in the absence of any commercial or financial relationships that could be construed as a potential conflict of interest.

## Publisher’s Note

All claims expressed in this article are solely those of the authors and do not necessarily represent those of their affiliated organizations, or those of the publisher, the editors and the reviewers. Any product that may be evaluated in this article, or claim that may be made by its manufacturer, is not guaranteed or endorsed by the publisher.

## Footnotes

1 Two pilot subjects received 6 and 15 blocks of training, respectively, before settling on the final 10 block training regimen.

2
http://www.python.org
