# Supplementary material for: Functional Plasticity Coupled With Structural Predispositions in Auditory Cortex Shape Successful Music Category Learning
Source: Front Neurosci. 2022 Jun 28;16:897239. doi: 10.3389/fnins.2022.897239 (PMC9274125; doi:10.3389/fnins.2022.897239)
Supplement: Supplementary file 1 [file Data_Sheet_1.pdf]

Supplementary Material

1 Supplementary analyses

1.1 Learning-related behavioral categorization changes

To determine whether pre- to post-test changes in identification slopes are different between the learning and control groups, we conducted a t-test on the probability density functions depicting the posttest – pretest slopes difference for each group (see **Supplementary Figure 1**). The distributions of the probability density differences indicate that the change in identification slopes differs between groups [$t_{(27)} = 2.43$, $p = 0.022$]. Consistent with the analyses reported in the main text (see "Behavioral Categorization Following Training"), the learning group achieved greater pre- to post-test identification slopes improvement than the control group.

2 Supplementary Figures

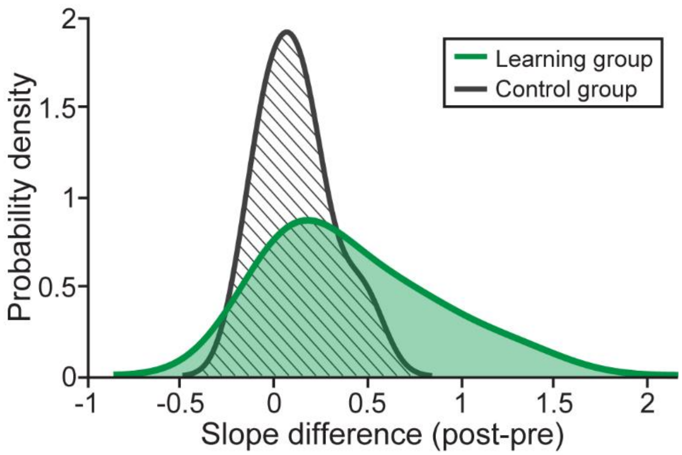

Supplementary Figure 1. Probability density functions comparing the (square-root transformed) posttest – pretest differences in identification slopes across groups suggest the improvement in categorization is greater for the learning group compared to the control group.

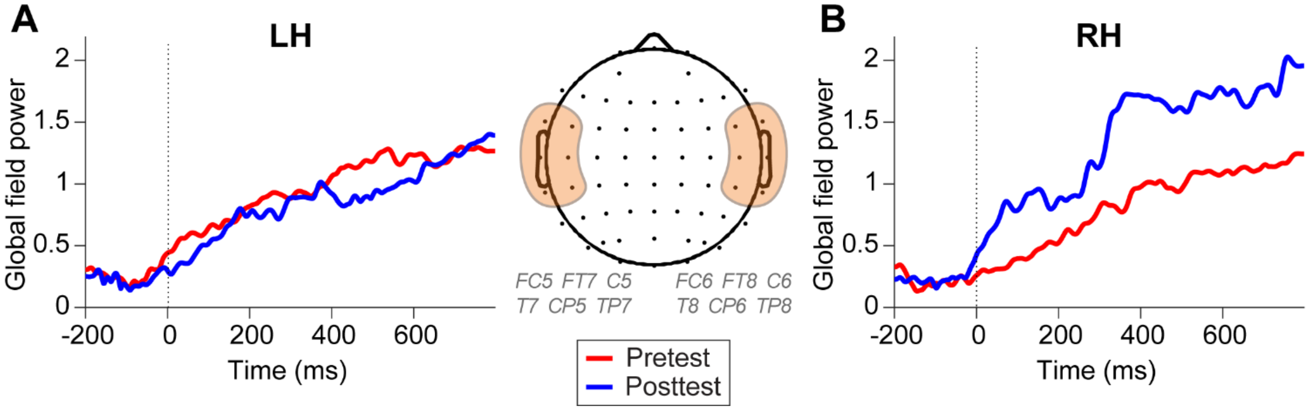

Supplementary Figure 2. Global field power indicates a greater pre- to post-test difference in neural activity over right hemisphere temporal electrodes (RH; **B**) compared to the left hemisphere (LH; **A**). These results support the conclusions reported in the main text using single-channel difference waveforms (i.e., T7 & T8; see "Electrophysiological Results" in main text); specifically, neuroplastic changes following musical interval category learning are biased towards the right hemisphere. Data shown for learning group only. (Center) Global field power was computed as the average activation across six electrodes over left and right temporal sites separately (i.e., LH: FC5, FT7, C5, T7, CP5, & TP7; RH: FC6, FT8, C6, T8, CP6, & TP8).

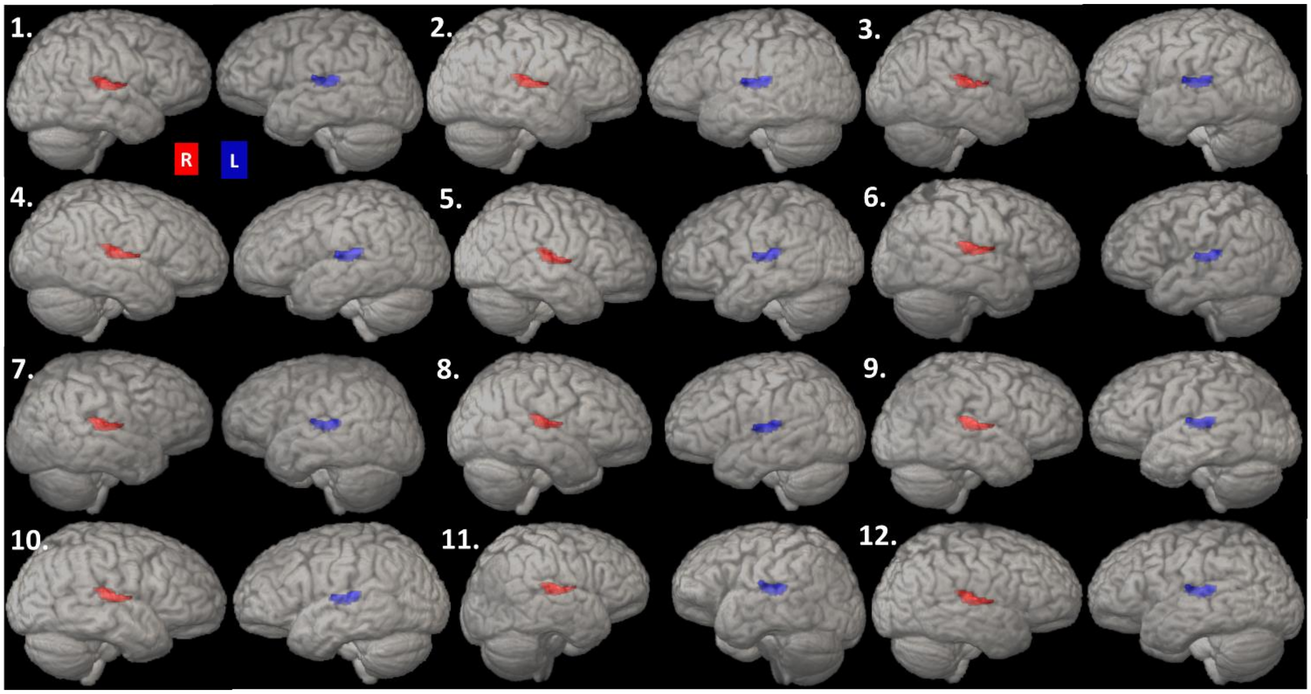

Supplementary Figure 3. Surface reconstruction maps of the MRI brain images depicting left and right Heschl's gyrus measured for each participant (blue and red, respectively). Each of the MRI images were registered to the AAL3 atlas using affine transformation, and the Heschl's gyrus mask was transformed to subject space for the segmentation. The surface reconstruction with the overlay was created in MRIcroGL 1.0 (University of South Carolina; www.nitrc.org/projects/mricrogl).

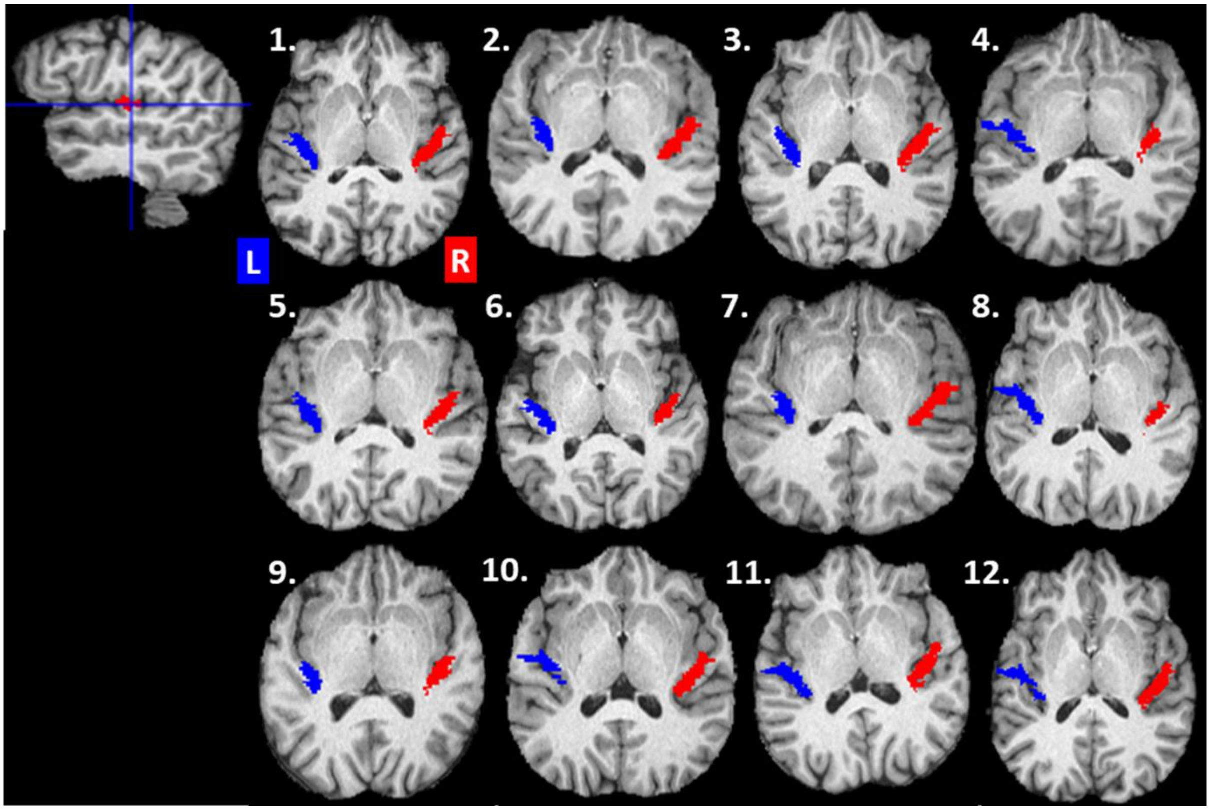

Supplementary Figure 4. Transverse slices through the supratemporal plane, rotated at an angle parallel with the Sylvian fissure (upper left), enable visualization of left and right Heschl's gyrus for each participant (blue and red, respectively). Image analysis was done using SPM12 in MATLAB (SPM; The Wellcome Centre for Human Neuroimaging, UCL Queen Square Institute of Neurology, London, UK).
